# On the Treatment of Psoriasis and Lepra Vulgaris

**Published:** 1850-03

**Authors:** 


*On the Treatment of Psoriasis and Lepra Vulgaris.* By M. Emery. M. Emery states, that when appointed to the St. Louis, several years ago, he tried all the various remedies so warmly recommended by authors, and of all these *arsenic* proved the best internal medicine; but besides the inconvenience which it sometimes gave rise to, its operation was very slow. The external use of *strong tar ointment* ($\frac{1}{3}$ to $\frac{1}{4}$ of tar) produces, in fact, a far more rapid cure than any other means. Of from 1500 to 1800 patients who have employed it, five sixths have been rapidly cured, and that without any ill consequence, or any greater frequency of relapse than after internal means. In 228 cases of psoriasis, the arsenic and tar were used conjointly, and 200 were cured within two months. Of all the preparations of *mercury*, the *protiodide* is alone efficacious ($\frac{1}{8}$); but it excites much irritation of the skin, and if applied to large surfaces, produces salivation. The *iodide of sulphur* is useful in psoriasis of the head; but if applied to large surfaces produces erysipelas. The conjoined use of the arsenic and tar-ointment constitutes, in fact, the best medication. In a disease so apt to recur, the greatest attention to diet is essential; and on the least symptom of recurrence, resort should be had to medicine, without waiting until it has become very bad. Before commencing, the patient takes a bath, and rubs in the ointment with gentle friction three times a day. In two or three days the strength of the ointment and activity of the friction are increased; and when the disease is of old standing, linen rags smeared with the ointment are to be kept applied. A tepid bath is taken once or twice a week. It is only in very irritable skins that the treatment is interrupted by the appearance of impetiginous pustules or furunculi. In about ten days we perceive, where the squamæ have fallen, a whitish circle circumscribing the patches, and extending from the circumference towards the centre. This is a sign of a decrease of the disease, which usually disappears in two or three months. With respect to the arsenic, we should begin with five drops of Fowler's solution, in four ounces of sugared water, which are to be divided into two doses. Every other day the dose is to be increased a drop, until twelve are reached. If we observe that the patches are becoming thinner, and acquiring a blackish-gray colour, this is a sign of saturation, and the dose is not to be increased; but even when this is not present, it is rarely proper to go beyond twelve or fifteen drops. Sometimes the skin becomes hot and painful around the patches; but this is relieved by tepid lotions, demulcent drinks, and diminution of the dose. After twelve or fifteen drops are reached, a feeling of constriction of the throat, or severe pain of the stomach, is perceived; and then the medicine should be suspended for a day or two, and recommenced *de novo* with the small dose. Pain near the heart, with palpitation, sometimes renders venesection requisite. If any contraction of the extensors of the limb is observed, the medicine must be at once abandoned; and as soon as the blackish-gray colour of the patches appears, which announces saturation and an approaching cure (though such spots may remain for months,) the arsenic must be discontinued.—*London Med. Gaz. from Comptes Rendus.*

